# Incidence, Risk Factors, and Effect on Allograft Survival of Glomerulonephritis Post-transplantation in a United Kingdom Population: Cohort Study

**DOI:** 10.3389/fneph.2022.923813

**Published:** 2022-07-14

**Authors:** Rute Aguiar, Elli Bourmpaki, Catey Bunce, Bola Coker, Florence Delaney, Leonardo de Jongh, Giovani Oliveira, Alistair Weir, Finola Higgins, Anastasia Spiridou, Syed Hasan, Jonathan Smith, Abdulrahim Mulla, Ben Glampson, Luca Mercuri, Rosa Montero, Maria Hernandez-Fuentes, Candice A. Roufosse, Naomi Simmonds, Menna Clatworthy, Adam McLean, Rutger Ploeg, Jim Davies, Kinga Anna Várnai, Kerrie Woods, Graham Lord, Rishi Pruthi, Cormac Breen, Paramit Chowdhury

**Affiliations:** ^1^ Department of Transplantation and Renal Medicine, Guy’s and St Thomas’ NHS Foundation Trust, London, United Kingdom; ^2^ School of Population Health and Environmental Sciences, Faculty of Life Sciences and Medicine, King’s College London, London, United Kingdom; ^3^ National Institute for Health and Care Research (NIHR) Biomedical Research Centre, Guy’s & St Thomas’ National Health Service (NHS) Foundation Trust and King’s College London, London, United Kingdom; ^4^ Data Research, Innovation and Virtual Environments Unit (DRIVE), Great Ormond Street Hospital for Children National Health Service (NHS) Foundation Trust, London, United Kingdom; ^5^ National Institute for Health and Care Research (NIHR) Imperial Biomedical Research Centre, Imperial College London and Imperial College Healthcare National Health Service (NHS) Trust, Hammersmith Hospital, London, United Kingdom; ^6^ Research Informatics Team, Imperial College Healthcare National Health Service (NHS) Trust, London, United Kingdom; ^7^ Tissue Remodelling Research at UCB, Slough, United Kingdom; ^8^ Department of Immunology and Inflammation, Imperial College London, London, United Kingdom; ^9^ Department of Medicine, University of Cambridge, Cambridge, United Kingdom; ^10^ Renal Section, Department of Medicine, Hammersmith Hospital Campus, Imperial College London, London, United Kingdom; ^11^ Nuffield Department of Surgical Sciences, Oxford Biomedical Research Centre, University of Oxford, Oxford, United Kingdom; ^12^ National Institute for Health and Care Research (NIHR) Oxford Biomedical Research Centre, Big Data Institute, University of Oxford, Oxford, Oxfordshire, United Kingdom; ^13^ Department of Computer Science, University of Oxford, Oxford, Oxfordshire, United Kingdom; ^14^ Oxford University Hospitals National Health Service (NHS) Foundation Trust, Oxford, Oxfordshire, United Kingdom; ^15^ Faculty of Biology Medicine and Health, University of Manchester, Manchester, United Kingdom

**Keywords:** kidney transplantation, recurrent glomerulonephritis, graft failure, machine learning, end-stage renal disease

## Abstract

**Background:**

Post-transplant glomerulonephritis (PTGN) has been associated with inferior long-term allograft survival, and its incidence varies widely in the literature.

**Methods:**

This is a cohort study of 7,623 patients transplanted between 2005 and 2016 at four major transplant UK centres. The diagnosis of glomerulonephritis (GN) in the allograft was extracted from histology reports aided by the use of text-mining software. The incidence of the four most common GN post-transplantation was calculated, and the risk factors for disease and allograft outcomes were analyzed.

**Results:**

In total, 214 patients (2.8%) presented with PTGN. IgA nephropathy (IgAN), focal segmental glomerulosclerosis (FSGS), membranous nephropathy (MN), and membranoproliferative/mesangiocapillary GN (MPGN/MCGN) were the four most common forms of post-transplant GN. Living donation, HLA DR match, mixed race, and other ethnic minority groups were associated with an increased risk of developing a PTGN. Patients with PTGN showed a similar allograft survival to those without in the first 8 years of post-transplantation, but the results suggest that they do less well after that timepoint. IgAN was associated with the best allograft survival and FSGS with the worst allograft survival.

**Conclusions:**

PTGN has an important impact on long-term allograft survival. Significant challenges can be encountered when attempting to analyze large-scale data involving unstructured or complex data points, and the use of computational analysis can assist.

## Introduction

Glomerulonephritidies are an important cause of end-stage kidney disease (ESKD), and their tendency to affect the younger age groups results in them being the most prevalent cause of ESKD in patients undergoing renal transplantation in the UK and the second most common in the USA (1, 2).

The incidence of recurrent glomerulonephritis (GN) post-transplant varies widely in studies (3, 4), reflecting a number of factors, including population characteristics, era of transplantation, biopsy practices, and duration of follow-up. Studies require large incident populations, and therefore previous studies have frequently utilized registry data (4, 5).

Recurrent GN is an important cause of allograft loss (6, 7) and accounts as the fourth most common cause in the UK (8). Graft survival for patients with a primary diagnosis of GN appears to be inferior to those with other diseases affecting the kidney (9), and patients who develop recurrent GN are twice as likely to lose their allograft compared with those who do not, with 45% losing their graft within 5 years of recurrence according to a registry-based study utilizing data over a 30-year period (5).

When conducting epidemiological studies using large-scale data, care needs to be taken regarding the accuracy of the clinical data points being considered. The use of computational analysis, such as text mining and natural language processing (NLP), allows the extraction of informative features from many types of raw and unstructured data, thus allowing greater accurately and standardisation. Such techniques may lead to more accurate results and conclusions (10, 11) and have already been successfully used in other fields to improve patient care (12, 13).

This multicenter study is the first in nephrology, utilizing computational analysis to aid in the diagnosis of GN and aiming to accurately estimate the incidence of GN post-transplantation in a United Kingdom cohort, analyse epidemiological factors influencing their emergence in the post-transplant period and comment on allograft outcomes.

## Materials and Methods

### Study Design, Setting, and Participants

This is a multicenter cohort study conducted in four UK transplant centres—Guy’s and St. Thomas’ NHS Foundation Trust, Cambridge University Hospitals NHS Foundation Trust, Oxford University Hospitals NHS Trust, and Imperial Healthcare NHS Trust.

The study was approved by the East Midlands Nottingham 2 Research Ethics Committee, with reference number 15/EM/0449.

The inclusion criteria for the patients during the study period were any individual (>16 years old) who has undergone kidney or simultaneous pancreas and kidney transplantation between January 2005 and October 2016 in the participating centres.

Only the first transplant episode per patient was considered during the time of study. There were no exclusion criteria.

### Clinical Data Collection

Routinely collected clinical data were extracted from electronic health records (EHRs) at each center, combined with data from NHS Blood & Transplant and aggregated in a data warehouse on a per transplant and personal level.

### Immunosuppression Regimens and Allograft Biopsy Practices Across Transplant Centers

Centers 1, 2, and 4 used mostly basiliximab as induction agent and the maintenance immunosuppression regimen of tacrolimus, mycophenolate (MMF), and prednisolone. Center 3 used a steroid-sparing protocol. Alemtuzumab was used as an induction agent in combination with tacrolimus monotherapy and a 7-day prednisolone regime. MMF was added in cases of *de novo* donor-specific antigens or high calculated reaction frequency. In the period of the study, all centers had their immunosuppression protocols unchanged except for center 1 that switched from cyclosporin to tacrolimus in 2010.

Regarding biopsy practices, center 3 had criteria-driven protocol biopsies at 1 year post-transplant. The other 3 centers only performed biopsies when clinically indicated.

### Text-Mining Biopsy Software and Its Validation

Software to extract the diagnoses of various glomerular diseases from unstructured biopsy text reports was developed using the open-source General Architecture for Text Engineering (14, 15) framework as the base. The validation process can be found in **Supplementary Appendix 1**.

### Statistical Analysis

Frequencies and percentages were used to present categorical factors and missing data. Means and standard deviations were used for normally distributed continuous data, while medians and interquartile ranges were used if their distribution was skewed. The distribution of continuous data was explored using histograms.

For the evaluation of possible risk factors for the diagnosis of GN post-transplantation, recipient-related characteristics were explored.

Univariate Cox regression analysis was used to explore the influence of each factor on time from transplant to the development of GN and time from transplant to graft failure. The category with the highest frequency was used as reference category in each exploratory factor. Factors with *P*-value <0.2 from their univariate Cox regression were added in the multiple Cox regression.

Kaplan–Meier plots were used for the survival analysis. For patients not presenting a graft failure, the date when their creatinine value was measured last was considered. A *P*-value of ≤0.05 was considered to indicate statistical significance.

All statistical analyses were performed with Stata/IC version 15.1.

## Results

### Characteristics of the Population Studied

During the period of the study, 7,623 patients had their first renal transplant. The population characteristics between centers were similar, except for an expected variation in ethnicity, with two of the four centres serving large multicultural urban areas. Only 45.9% of the patients had information regarding the cause of ESKD. Of these, in 38.1% of cases, the cause of ESKD was unknown, while 11.1% had a diagnosis of a specific GN and 3.2% had a diagnosis of chronic glomerulonephritis of an unspecified nature.

The demographics of our population and patients’ transplant characteristics are described in **Table 1**.

**Table 1:** Demographic and transplant characteristics of patients who had their first kidney transplant during the period 2005–2016 by center.

Baseline and transplant characteristics		Centers				
		Center 1	Center 2	Center 3	Center 4	Total
		*N* = 1,694 (22.2% of *T*)*n* (% of *N*)	*N* = 2,270 (29.8% of *T*)*n* (% of *N*)	*N* = 1,741 (22.8% of *T*) *n* (% of *N*)	*N* = 1,918 (25.2% of *T*) *n* (% of *N*)	*T* = 7,623 (100.0%)*n* (% of *T*)
Gender	Female	620 (36.6)	897 (39.5)	638 (36.6)	732 (38.2)	2,887 (37.9)
	Male	1,074 (63.4)	1,373 (60.5)	1,102 (63.3)	1,186 (61.8)	4,735 (62.1)
	Unknown	–	–	1 (0.1)	–	1 (0.0)
Age at date of transplant	Years, mean, SD, (range), *n*	48.8 (13.5) (17.0, 76.0) *n* = 1,694	47.1 (13.7) (16.0, 79.0) *n* = 2,270	49.5 (12.9) (18.0, 78.0) *n* = 1,741	48.2 (12.6) (17.0, 81.0) *n* = 1,918	48.3 (13.2) (16.0, 81.0) *n* = 7,623
Age groups at date of transplant	16–30	185 (10.9)	294 (13.0)	168 (9.6)	163 (8.5)	810 (10.6)
	31–45	487 (28.7)	722 (31.8)	478 (27.5)	662 (34.5)	2,349 (30.8)
	46–55	434 (25.6)	583 (25.7)	483 (27.7)	532 (27.7)	2,032 (26.7)
	≥56	588 (34.7)	671 (29.6)	612 (35.2)	561 (29.2)	2,432 (31.9)
Ethnicity^a^	White	1,415 (83.5)	1,587 (69.9)	782 (44.9)	1,558 (81.2)	5,342 (70.1)
	Mixed	7 (0.4)	32 (1.4)	21 (1.2)	–	60 (0.8)
	Asian or Asian British	60 (3.5)	135 (5.9)	465 (26.7)	252 (13.1)	912 (12.0)
	Black or Black British	171 (10.1)	371 (16.3)	266 (15.3)	77 (4.0)	885 (11.6)
	Other ethnic groups	12 (0.7)	58 (2.6)	142 (8.2)	25 (1.3)	237 (3.1)
	Not stated	29 (1.7)	87 (3.8)	65 (3.7)	6 (0.3)	187 (2.5)
Blood group	Missing	–	–	11 (0.6)	–	11 (0.1)
	A	711 (42.0)	858 (37.8)	587 (33.7)	817 (42.6)	2,973 (39.0)
	AB	62 (3.7)	107 (4.7)	97 (5.6)	104 (5.4)	370 (4.9)
	B	168 (9.9)	293 (12.9)	331 (19.0)	241 (12.6)	1,033 (13.6)
	0	753 (44.5)	1,012 (44.6)	715 (41.1)	756 (39.4)	3,236 (42.5)
Comorbidities	Cardiovascular disease	28 (1.7)	183 (8.1)	5 (0.3)	–	216 (2.8)
	Diabetes	72 (4.3)	485 (21.4)	19 (1.1)	–	576 (7.6)
	Current smoker	3 (0.2)	89 (3.9)	–	–	92 (1.2)
	Ex-smoker	–	55 (2.4)	–	–	55 (0.7)
	Hypertension	1,316 (77.7)	1,406 (61.9)	4 (0.2)	–	2,726 (35.8)
Donor type	DBD	530 (31.3)	887 (39.1)	791 (45.4)	982 (51.2)	3,190 (41.8)
	DCD	740 (43.7)	428 (18.9)	197 (11.3)	467 (24.3)	1,832 (24.0)
	Living—related	243 (14.3)	571 (25.2)	408 (23.4)	268 (14.0)	1,490 (19.5)
	Living—unrelated	181 (10.7)	384 (16.9)	345 (19.8)	201 (10.5)	1,111 (14.6)
Donor’s age^b^	Years, mean, SD (range), *n*	–	46.4 (15.4) (0.0, 83.0) *n* = 2,270	54.8 (14.8) (11.0, 90.0) *n* = 1,741	45.5 (16.0) (1.0, 85.0) *n* = 1,918	48.5 (16.0) (0.0, 90.0) *n* = 5,959
Donor’s gender	Missing	–	1 (0.0)	–	–	1 (0.0)
	Female	786 (46.4)	1,150 (50.7)	936 (53.8)	923 (48.1)	3,795 (49.8)
	Male	908 (53.6)	1,119 (49.3)	805 (46.2)	995 (51.9)	3,827 (50.2)
Years of transplantation	2005–2010	787 (46.5)	966 (42.6)	904 (51.9)	858 (44.7)	3,515 (46.1)
	2011–2016	907 (53.5)	1,304 (57.4)	837 (48.1)	1,060 (55.3)	4,108 (53.9)
Cold ischemia time	Hours, median (IQR), *n*	12.7 (7.0, 16.2) *n* = 1,658	10.8 (4.0, 15.1) *n* = 2,009	12.0 (3.0, 19.0) *n* = 1,710	12.0 (9.0, 15.0) *n* = 1,656	12.0 (4.0, 16.0) *n* = 7,033
	Missing	36 (2.1)	261 (11.5)	31 (1.8)	262 (13.7)	590 (7.7)
HLA mismatch A	0	336 (19.8)	491 (21.6)	354 (20.3)	401 (20.9)	1,582 (20.8)
	1	876 (51.7)	1,192 (52.5)	867 (49.8)	1,025 (53.4)	3,960 (51.9)
	2	482 (28.5)	559 (24.6)	519 (29.8)	491 (25.6)	2,051 (26.9)
	Missing	–	28 (1.2)	1 (0.1)	1 (0.1)	30 (0.4)
HLA mismatch B	0	249 (14.7)	353 (15.6)	264 (15.2)	256 (13.3)	1,122 (14.7)
	1	1,087 (64.2)	1,342 (59.1)	1,091 (62.7)	1,079 (56.3)	4,599 (60.3)
	2	358 (21.1)	547 (24.1)	385 (22.1)	582 (30.3)	1,872 (24.6)
	Missing	–	28 (1.2)	1 (0.1)	1 (0.1)	30 (0.4)
HLA mismatch DR	0	639 (37.7)	771 (34.0)	644 (37.0)	627 (32.7)	2,681 (35.2)
	1	840 (49.6)	1,118 (49.3)	869 (49.9)	972 (50.7)	3,799 (49.8)
	2	215 (12.7)	353 (15.6)	227 (13.0)	318 (16.6)	1,113 (14.6)
	Missing	–	28 (1.2)	1 (0.1)	1 (0.1)	30 (0.4)
HLA mismatch A/B/DR	0–2	591 (34.9)	772 (34.0)	587 (33.7)	547 (28.5)	2,497 (32.8)
	3–4	904 (53.4)	1,188 (52.3)	934 (53.6)	1,142 (59.5)	4,168 (54.7)
	5–6	199 (11.7)	282 (12.4)	219 (12.6)	228 (11.9)	928 (12.2)
	Missing	–	28 (1.2)	1 (0.1)	1 (0.1)	30 (0.4)
ESKD cause captured	No	802 (47.3)	960 (42.3)	447 (25.7)	1,918 (100.0)	4,127 (54.1)
	Yes	892 (52.7)	1,310 (57.7)	1,294 (74.3)	–	3,496 (45.9)

IQR, interquartile range (25 to 75% quartiles).

a Ethnicity was coded according to the “Ethnic Category Code” of NHS; for more details, please see the link below:

http://www.datadictionary.nhs.uk/data_dictionary/attributes/e/end/ethnic_category_code_de.asp?shownav=1.

b Results from CUH available for 30 patients. Missing data from CUH: 1,664 (98.2%).

### Diagnosis of GD Post-transplantation

During the period of the study, 277 (3.6%) patients presented with evidence of glomerular disease in their allograft biopsy.

The histological findings in the renal allograft were IgA nephropathy (IgAN) in 48.7% of the biopsies, focal segmental glomerulosclerosis (FSGS) in 18.1%, membranous nephropathy (MN) in 7.2%, membranoproliferative GN and mesangiocapillary GN (MPGN/MCGN) in 2.9%, minimal change disease (MCD) in 0.4%, and thrombotic microangiopathy (TMA) in 22.7%.

A statistical analysis was carried out using the four most common GNs, namely, IgAN, FSGS, MN, and MPGN/MCGN. TMA represents a heterogeneous group of conditions and often cannot be unequivocally attributed to a single underlying etiology (16) even after an in-depth clinico-pathological correlation, and hence this group was excluded at this stage from further analysis. Therefore, the percentage of post-transplant GN (IgAN, FSGS, MN, MPGN, and MCD) found in allografts in the four centers participating in this study was 2.8%.

Between 24 and 32 patients per 1,000 transplanted patients (95% CI) are estimated to develop a GN post-transplantation (**Table 2**).

**Table 2:** Incidence estimates by post-transplant glomerulonephritis (GN) (N = 214).

Post-transplant GD	Incidence estimate (per 1,000 patients) (95% CI)
	Total *N* = 7,623
IgA nephropathy	17.71 (14.87 to 20.93)
FSGS	6.56 (4.87 to 8.64)
Membranous nephropathy	2.62 (1.60 to 4.05)
MPGN	1.05 (0.45 to 2.07)
MCD	0.13 (0.00 to 0.73)
All patients who developed any GN^a^	28.07 (24.48 to 32.03)

FSGS, focal segmental glomerulosclerosis; aHUS, atypical hemolytic uremic syndrome; MPGN, membranoproliferative glomerulonephritis; MCD, minimal change disease.

a Any glomerulonephritis disease: IgAN, FSGS, MPGN, MN, or MCD.

The median time from transplant to histopathological diagnosis of GN was 701.5 days (IQR: 168–1,742).

### Risk Factors for the Development of Post-transplant GN

Younger age groups, 16–30 years old and 31–45 years old, were found to have a 1.7 (95% CI: 1.113–2.635; *P*-value: 0.014) and 1.46 (95% CI: 1.027–2.078; P-value: 0.035) increased risk for developing a GN in their allograft, respectively.

Living related donation was identified to increase the risk for the development of a GN (HR: 1.74; 95% CI: 1.238–2.445; *P*-value: 0.001) as well as unrelated donation (HR: 1.3; 95% CI: 1.042–2.264; *P*-value: 0.03).

Protective factors against the development of GN in the allograft were identified as female gender (HR: 0.70; 95% CI: 0.521–0.933; *P*-value: 0.015), black ethnicity (HR: 0.54; 95% CI: 0.318–0.923; *P*-value: 0.024), transplantation period from 2005 to 2010 (HR: 0.64; 95% CI: 0.47–0.864; *P*-value: 0.004), and cold ischemia time (HR: 0.98; 95% CI: 0.966–1; *P*-value: 0.05) (**Table 3**).

**Table 3:** Risk factors for the development of post-transplant glomerulonephritis (IgAN, FSGS, MPGN, MN, and MCD) (N = 7,560).

Characteristics		UnivariateHR (95% CI)	*P*-value	MultivariableHR (95% CI)	*P*-value
Gender	Female	0.697 (0.521–0.933)	0.015	0.754 (0.556–1.023)	0.069
	Male	1		1	
Age groups at date of transplant	16–30	1.712 (1.113–2.635)	0.014		
	31–45	1.461 (1.027–2.078)	0.035		
	46–55	1.069 (0.720–1.586)	0.741		
	≥56	1			
Age at date of transplant		0.985 (0.975–0.995)	0.004	0.988 (0.977–0.999)	0.036
Ethnic groups	White	1		1	
	Black	0.542 (0.318–0.923)	0.024	0.556 (0.319–0.971)	0.039
	Asian	1.204 (0.838–1.729)	0.316	1.288 (0.888–1.867)	0.182
	Other	1.584 (0.957–2.621)	0.073	1.714 (1.027–2.861)	0.039
Blood group	A	0.889 (0.654–1.207)	0.45	–	
	AB	1.288 (0.747–2.22)	0.362	–	
	B	0.914 (0.606–1.377)	0.666	–	
	O	1		–	
Donor type	DBD	1		1	
	DCD	1.244 (0.835–1.854)	0.283	1.351 (0.892–2.046)	0.155
	Living—related	1.74 (1.238–2.445)	0.001	1.777 (0.990–3.192)	0.054
	Living—unrelated	1.536 (1.042–2.264)	0.03	2.136 (1.165–3.915)	0.014
Donor’s gender	Female	1.208 (0.923–1.581)	0.169	1.195 (0.897–1.593)	0.224
	Male	1		1	
Donor’s age		1.005 (0.996–1.015)	0.281	–	
Year of transplantation	2005–2010	0.637 (0.47–0.864)	0.004	0.596 (0.430–0.824)	0.002
	2011–2016	1		1	
Cold ischemia time		0.983 (0.966–1)	0.05	1.010 (0.980–1.042)	0.504
HLA mismatch A	0	1.047 (0.747–1.467)	0.79	–	
	1	1		–	
	2	0.977 (0.708–1.348)	0.887	–	
HLA mismatch B	0	1.268 (0.894–1.799)	0.182	1.132 (0.757–1.692)	0.545
	1	1		1	
	2	0.959 (0.684–1.344)	0.806	0.941 (0.638–1.388)	0.759
HLA mismatch DR	0	1.316 (0.987–1.754)	0.062	1.399 (1.007–1.942)	0.045
	1	1		1	
	2	1.078 (0.707–1.643)	0.728	1.054 (0.653–1.701)	0.829
HLA mismatch A/B/DR	0–2	1.194 (0.893–1.597)	0.232	–	
	3–4	1		–	
	5–6	1.21 (0.805–1.819)	0.359	–	

HR, hazard ratio.

From the adjusted analysis, living donation was shown to be a risk factor for developing a GN post-transplantation, with living unrelated donation associated with a risk increase of 2.1 times (95% CI: 1.171–3.928; *P*-value: 0.013) and living related donation with a risk increase of 1.8 times (95% CI: 1.007–3.264; *P*-value: 0.047). Ethnicity, namely, mixed race and other ethnic minority groups, was identified as another risk factor for post-transplant GN (HR: 1.7; 95% CI: 1.038–2.892; *P*-value: 0.035). An HLA DR mismatch of 0 was also a risk factor for developing a GN after transplantation, with an associated 1.4 times increase in risk (95% CI: 1.006–1.94; *P*-value: 0.046).

The protective factors against the development of GN in the allograft were also identified, specifically, black race (HR: 0.56; 95% CI: 0.319–0.972; *P*-value: 0.039) and year of transplantation between 2005 to 2010 (HR: 0.6; 95% CI: 0.429–0.823; *P*-value: 0.002).

### Post-transplant GN, Allograft, and Patient Survival

Patients who developed GN post-transplantation presented a median allograft lifespan of 1,207 days (IQR: 365.0–2,208.0). Allograft failure was observed in 25% (54 patients) with a median time from the histopathological diagnosis of GN to failure of 224 days (IQR: 17–414). Death occurred in 8.9% (19 patients) at 587 days (IQR: 204.0–1,223.0) following the diagnosis of GN in the allograft.

From the adjusted analysis, the year of transplantation between 2005 and 2010 was shown to be the only risk factor for graft failure, with an increased risk of 1.4 times (95% CI: 1.14–1.61; *P*-value: 0.001) (**Table 4**). Donation after cardiac death was identified as a protective factor against graft failure (HR: 0.79; 95% CI: 0.65–0.982; *P*-value: 0.033).

**Table 4:** Risk factors for graft loss (N = 7,560).

Characteristics		UnivariateHR (95% CI)	*P*-value	MultivariableHR (95% CI)	*P*-value
Gender	Female	0.942 (0.833–1.065)	0.342	-	
	Male	1		-	
Age groups at date of transplant	16–30	0.630 (0.508–0.780)	<0.001	–	–
	31–45	0.685 (0.592–0.794)	<0.001	–	
	46–55	0.711 (0.609–0.830)	<0.001	–	
	≥56	1		–	
Age at date of transplant		1.014 (1.01–1.019)	<0.001	1.013 (1.007–1.020)	0.000
Ethnic groups	White	1		1	
	Black	0.920 (0.764–1.109)	0.384	0.914 (0.740–1.130)	0.401
	Asian	0.696 (0.573–0.847)	<0.001	0.659 (0.530–0.819)	0.000
	Other	0.704 (0.512–0.968)	0.031	0.760 (0.545–1.060)	0.106
Blood group	A	1.034 (0.909–1.177)	0.605	1.010 (0.866–1.178)	0.899
	AB	0.741 (0.547–1.003)	0.052	0.716 (0.504–1.017)	0.062
	B	0.760 (0.625–0.923)	0.006	0.887 (0.708–1.110)	0.294
	O	1		1	
Donor type	DBD	1		1	
	DCD	0.975 (0.835-1.136)	0.745	0.805 (0.655–0.989)	0.039
	Living—related	0.583 (0.493–0.689)	<0.001	0.408 (0.302–0.525)	0.000
	Living—unrelated	0.609 (0.506–0.734)	<0.001	0.338 (0.251–0.456)	0.000
Donor’s gender	Female	1.036 (0.920–1.166)	0.552	–	
	Male	1		–	
Donor’s age		1.007 (1.004–1.012)	<0.001	1.007 (1.002–1.011)	0.009
Year of transplantation	2005–2010	1.282 (1.110–1.479)	0.001	1.355 (1.141–1.61)	0.001
	2011–2016	1		1	
Cold ischemia time		1.016 (1.009–1.023)	<0.001	0.974 (0.961–0.988)	0
HLA mismatch A	0	0.920 (0.788–1.075)	0.297	–	
	1	1		–	
	2	1.041 (0.906–1.196)	0.566	–	
HLA mismatch B	0	0.898 (0.756–1.068)	0.226	–	
	1	1		–	
	2	0.966 (0.837–1.114)	0.636	–	
HLA mismatch DR	0	0.988 (0.868–1.125)	0.862	–	
	1	1		–	
	2	0.967 (0.807–1.157)	0.715	–	
HLA mismatch A/B/DR	0–2	0.917 (0.805–1.044)	0.194	0.911 (0.778–1.067)	0.246
	3–4	1		1	
	5–6	0.872 (0.722–1.053)	0.157	1.132 (0.900–1.424)	0.291

HR, hazard ratio.

**Figure 1:**
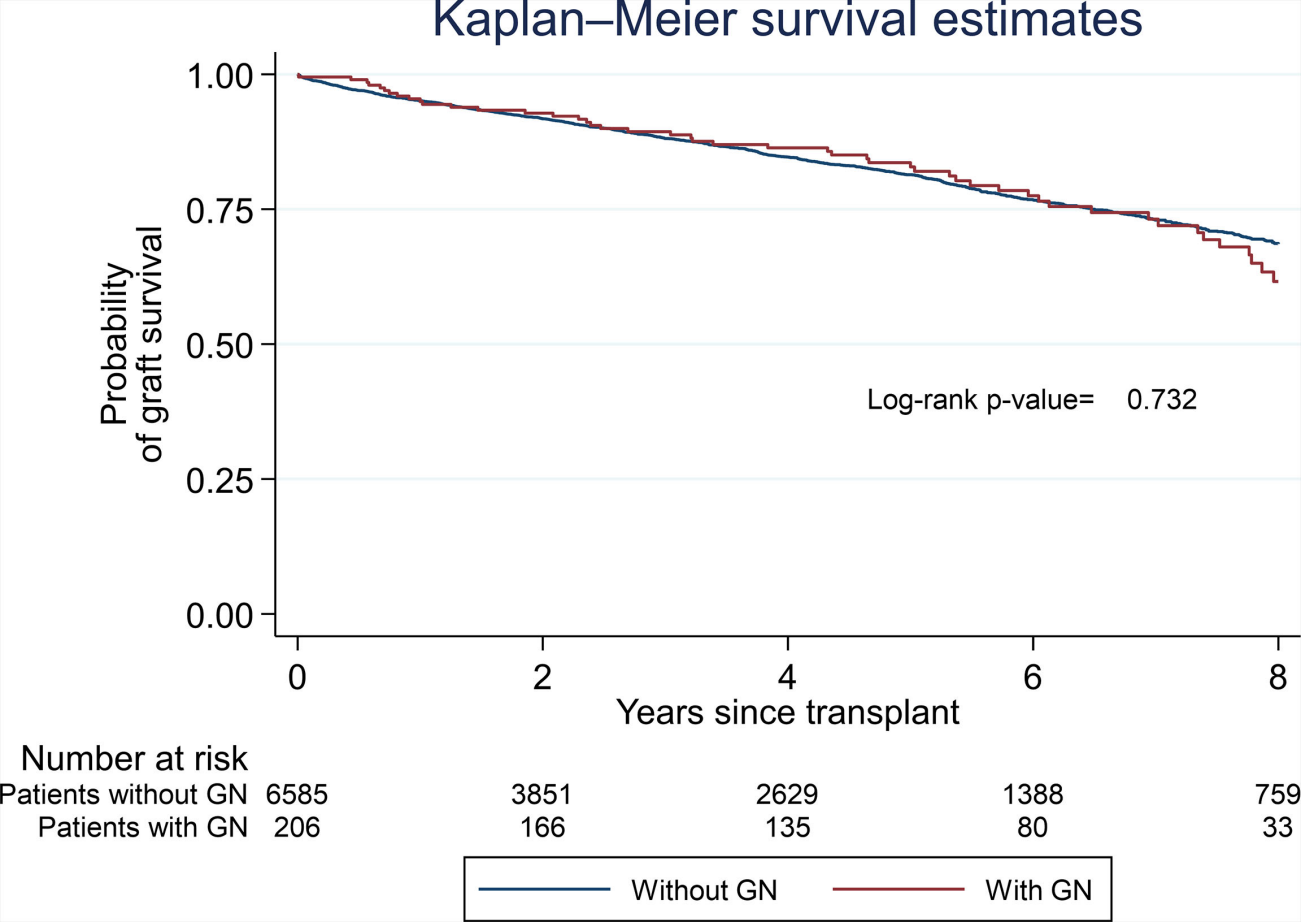
Graft survival for patients with and without a post-transplant glomerulonephritis (*N* = 7,560).

When comparing the survival estimate in patients who developed any type of GN post-transplantation to patients who did not develop a disease, the former group of patients was shown to have a similar allograft survival until after approximately 7.5–8 years post-transplantation, at which point there is a suggestion that they did less well (**Figure 1**).

The probability of graft survival for patients who developed a diagnosis of GN after transplantation, regardless of its histopathological pattern, was approximately 95% at 1 year (95% CI: 0.91–0.97) and 83% at 5 years (95% CI: 0.76–0.88).

**Figure 2:**
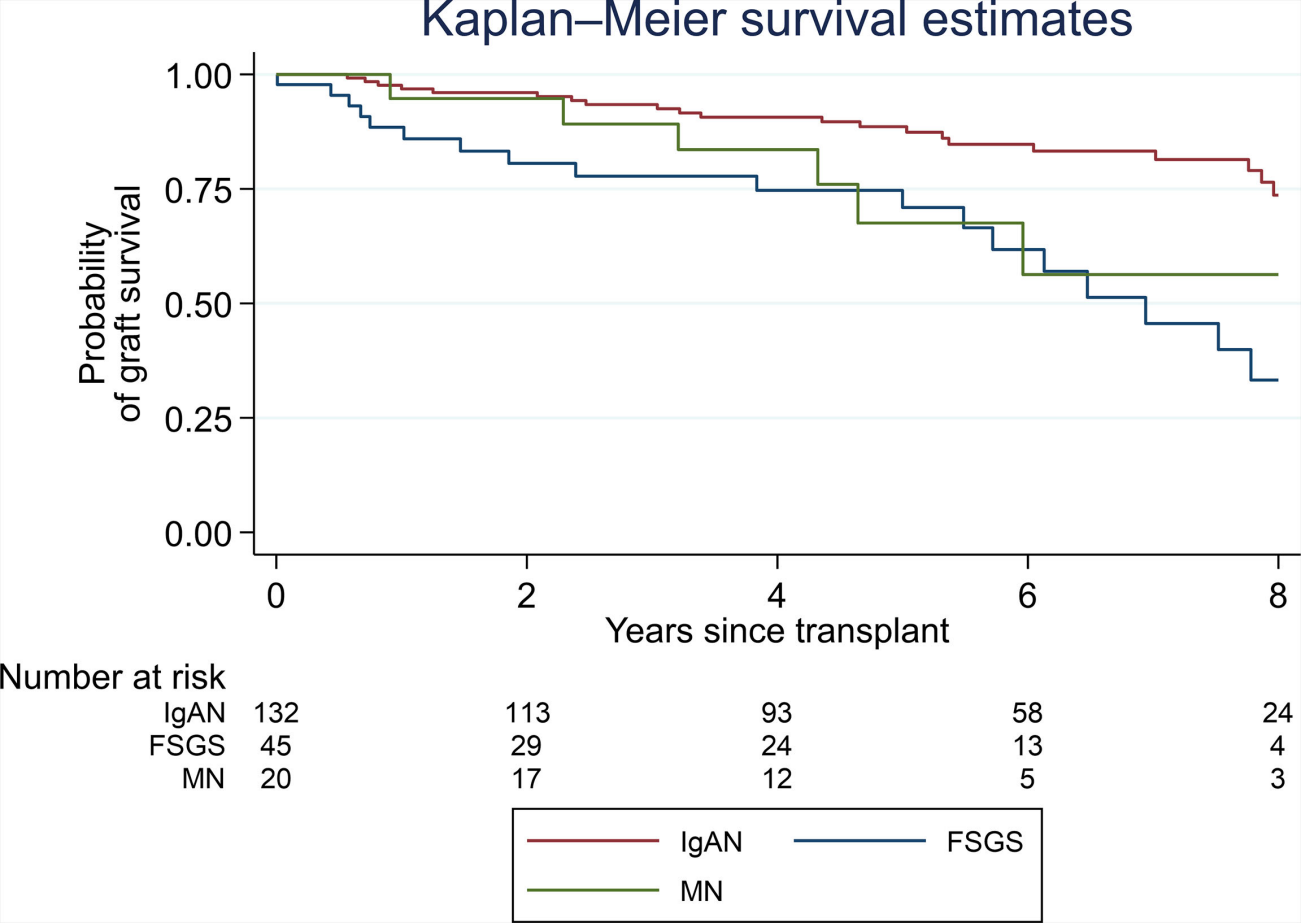
Kaplan–Meier survival estimates of post-transplant glomerulonephritis by histopathological type.

**Figure 2** shows the survival analysis plotted by the subtypes of GN. Only IgAN, FSGS, and MN are presented, as MPGN and MCD presented small frequencies to deliver meaningful comparisons.

IgAN demonstrated the most favorable outcome showing a small and stable decline in allograft survival until 8 years post-transplant, with an allograft survival of approximately 75% at this time point. In contrast, FSGS experienced the worst outcome with a progressive decline post-transplant, showing an approximate allograft survival of 30% at 8 years post-transplant. MN presented good allograft survival in the first year post-transplant, with an allograft survival of approximately 55–60% at 7 years post-transplant.

## Discussion

This is the first large-scale UK study investigating the incidence and effects of post-transplant GN. It is one of the first reports detailing the use of computational techniques to aid in the analysis of renal biopsy reports in a large patient cohort across multiple centers. We found the overall incidence of GN after renal transplantation to be 2.8%. A large study using registry data focused on recurrent disease and found that the recurrence of the four most common GN subtypes was around 10% (5). However, a comparison between studies is complicated by variation and accuracy of defining the at-risk denominator population and is one of the factors explaining the wide variations.

A major limitation of this study was the inability to accurately define the cause of ESRD in a large proportion of patients and prevented us commenting specifically on recurrent disease. As well as a significant amount of missing data (46%), even where data was available, a large proportion of patients with ESKD had no determined cause (41%). This is particularly true in black patients where a biopsy is less likely to occur and ESKD ascribed to hypertension (17). The high proportion of non-Caucasian patients in our study might explain why only 11.1% of our transplanted population had GN as the cause of ESKD, which was lower than those of other studies. The cause of ESRD is a complex data point requiring a careful clinico-pathological correlation and even when coded is prone to inaccuracy in large registry datasets. For these reasons, we felt that attempts to comment on the rates of recurrence by considering at risk groups determined by the cause of ESKD would be fraught with inaccuracy in our study population.

Another factor why our study has highlighted a low rate of post-transplant GN might relate to biopsy practice and the low frequency of protocol biopsies carried out. Only one center carried out a form of protocol biopsy at 1 year post-transplant based on specific criteria such as low eGFR. However, the practice of early protocol biopsy is likely to have a little effect as most post-transplant GN develops after the first year, at least histologically. The criteria for performing a biopsy in the longer term vary widely not just between centers but also between individual clinicians and has also changed over time, with the understanding that there are multiple causes of long-term allograft loss.

In common with other studies, we found IgAN as the most common GN found in post-transplant biopsies (6, 18). The incidence of post-transplant FSGS is likely to be under-estimated as recurrent FSGS often presents early and is diagnosed clinically in the first instance due to increasing proteinuria in a patient with a primary FSGS as the known cause of ESKD. A diagnosis of recurring MCGN/MPGN in an allograft can be challenging from a histological perspective due to the recent change in classification, thus reflecting a better understanding of its subtypes (19, 20).

Living donation has been identified as a risk factor for developing a post-transplant GN, however with conflicting results (5, 21, 22). In our study, living donation, both related and unrelated, was found to be a risk factor in univariate and subsequent adjusted analysis. Younger age at transplantation was found to be a risk factor in univariate analysis in keeping with the findings of Allen et al., likely reflecting an increased prevalence of GN as the cause of ESKD as well as an increased likelihood of receiving living donor kidney.

Black race was identified as a protective variable for the development of post-transplant GN. This finding could be explained by the fact that this group is more greatly affected by other diseases involving the kidneys (hypertension and sickle cell disease) or because some forms of GN related to APOL1 genotype have a very low risk of recurrence (23). It has not been previously mentioned in the literature, possibly because of the low representation of this group in previous studies. Mixed race and other ethnic minority groups were identified as a risk factor for post-transplant GN, though this finding was unexpected; it is of unclear significance without more granular data.

With regards to immunosuppression and its role in the development or reduction of GN disease in the renal allograft (24, 25), we were unable to assess this due to the lack of data on immunosuppression at an individual patient level. However, we chose a study period during which no major changes in immunosuppression occurred, with standard therapy being basiliximab induction followed by maintenance therapy with tacrolimus, mycophenolate mofetil, and prednisolone. We were unable to assess the effect of steroid withdrawal which has been suggested as a risk factor for the development of recurrent IgA nephropathy. Of note is that we are indirectly able to comment on induction therapy in that three out of the four centers in our study routinely used IL-2 receptor blocker therapy while one routinely used lymphocyte depletion, and no center effect was noted in the development of post-transplant GD.

Cold-ischemia time and HLA mismatches have been implied in the development of GN in the allograft (5, 26). There was no independent effect of total cold-ischemic time observed in our study. Interestingly, a mismatch of 0 at the HLA-DR locus was found to be an independent risk factor for developing GN after transplantation. This could be explained by the indirect effect on episodes of rejection—increasing allograft survival hence increasing time to develop post-transplant GN. However, this finding needs to be analyzed with caution as it could be explained by other causes, such as unknown genetic predisposition to certain GN types.

In our study, allograft failure was observed in 25% of the patients who developed a GN, regardless of histopathologic type. This percentage is in line with previous studies (5, 27).

In our cohort, patients with any type of post-transplant GN presented a similar allograft survival to patients who did not develop the disease until after approximately 7.5-8 years post-transplantation, at which point there is a suggestion that they did less well. Allograft survival at 1 year was 95% and at 5 years was 83%. Recipients with allograft GN also presented a lower allograft survival when compared with the allograft survival estimate for all UK centers (2) since allograft survival for deceased donor and living donor at 1 year was 95 and 98% and at 5 years was 87 and 93%, respectively.

From all the variables studied, year of transplantation between 2005 and 2010 was shown to be the only risk factor for graft failure. Whether this is due to the immunosuppression used in the early era (28), lower biopsy practices across time and thus possibly missing the disease, or the number of events of alloimmune rejection, we do not know since the latter factor was not included in the study.

The different types of GN, both in native and transplanted kidneys, behave in distinct ways. As in other studies (5), we found that IgAN demonstrates the best overall prognosis. IgAN post-transplant has been recognized as having a high rate of recurrence, especially when a longer time post-transplant is taken in consideration and there is a lower rate of allograft loss (29). Previous studies have suggested that MCGN/MPGN recurrence has the worst prognosis (5, 7), but we did not have sufficient numbers in this category to consider long-term survival. In MN, the allograft survival curve appears stable in the first year post-transplant; however, this might be due to a possible delay in histopathologic diagnosis since early recurrent MN often does not include the classical histopathologic features (19, 30). In our study, FSGS experienced the worst outcome with an approximate allograft survival of 30% at 8 years post-transplant. This finding is similar to the results shown in a recent multicenter study, especially in patients with recurrent FSGS and a partial or no response to treatment (31). Differences in survival estimates in these latter GNs were also highlighted by other groups, with risk of graft failure being significantly higher in recipients with FSGS and MN (7, 32).

A major strength of our study is the direct analysis of biopsy reports to determine post-transplantation GN rather than relying on registry data, thus reducing potential inaccuracy in data gathering or disease coding.

The increasing use of EHRs provides an opportunity for the automated collection and analysis of large patient cohorts and “big data” which is of particular importance in nephrology, where the incidence of specific diseases is rare. Our study highlights the difficulties in standardization and accurate collection of complex data points, such as the diagnosis of renal disease, which rely on the analysis of unstructured data. We demonstrate that computational analysis has potential for use in this area, with more advanced techniques involving NLP and artificial intelligence offering the potential of fully automated extraction of complex data points, such as the diagnosis of renal disease. Other limitations to our study are the type of study, retrospective, as well as the abovementioned absence of cause of ESKD, which did not allow us to differentiate recurrent from *de novo* disease post-transplantation. An improved understanding of the factors limiting long-term renal allograft survival is of importance, both in terms of counselling patients and informing further study to improve long-term allograft survival. Our study adds to the body of knowledge to this area. The identification of highly accurate cohorts of patients with specific GNs in their allografts will allow future detailed studies within these groups. As most GNs have an immunological basis, patients with post-transplant GN occurring despite the use of immunosuppression may provide opportunity to study an extreme clinical phenotype to better understand the contribution of genetic components.

In conclusion, in this large-scale UK study, we found that GN post-renal transplantation has an important impact on long-term allograft survival, and significant challenges can be encountered when attempting to analyze large-scale data. Nonetheless, machine learning can aid in the study of complex data points.

## Data Availability Statement

The original contributions presented in the study are included in the article/**Supplementary Material**. Further inquiries can be directed to the corresponding author.

## Ethics Statement

The studies involving human participants were reviewed and approved by the East Midlands Nottingham 2 Research Ethics Committee with reference number 15/EM/0449. Written informed consent for participation was not required for this study in accordance with the national legislation and the institutional requirements.

## Author Contributions

The research idea and study design were contributed by PC, RA, CB, MC, and RM. EB, BC, FD, LJ, GO, SH, AMu, BG, LM, JD, KV, and KW contributed to the analysis and interpretation of data. JS and BC contributed to the development of the text-mining biopsy software. Statistical analysis was done by EB and CB. AW, FH, and AS contributed to the management of the project. Drafting the article and providing intellectual content were done by RA, PC, EB, CB, CR, NS, CB, and RP. Approval of the submitted version of the manuscript was done by RA, EB, CB, PC, RP, CB, MH-F, CR, NS, AMc, RP, and GL. Supervision and mentorship was provided by PC. All authors contributed to the article and approved the submitted version.

## Funding

This research was funded by the National Institute for Health Research (NIHR) Biomedical Research Centre based at Guy’s and St. Thomas’ NHS Foundation Trust and King’s College London. The views expressed are those of the author(s) and not necessarily those of the NHS, the NIHR, or the Department of Health.

## Conflict of Interest

MH-F is currently an employee of UCB Celltech, a pharmaceutical company. Her involvement in the conduct of this research was solely in her capacity as academic at King’s College London.

The remaining authors declare that the research was conducted in the absence of any commercial or financial relationships that could be construed as a potential conflict of interest.

## Publisher’s Note

All claims expressed in this article are solely those of the authors and do not necessarily represent those of their affiliated organizations, or those of the publisher, the editors and the reviewers. Any product that may be evaluated in this article, or claim that may be made by its manufacturer, is not guaranteed or endorsed by the publisher.

## References

[1] Saran R Robinson B Abbott KC Agodoa LYC Bragg-Gresham J Balkrishnan R. US Renal Data System 2018 Annual Data Report: Epidemiology of Kidney Disease in the United States. Am J Kidney Dis Off J Natl Kidney Foundation (2019) 73(3 Suppl 1):A7–8. doi: 10.1053/j.ajkd.2019.01.001 PMC6620109 30798791

[2] UK Renal Registry. UK Renal Registry 21st Annual Report – Data to 31/12/2017. Bristol, UK: UKRR of The Renal Association (2019). Available at: https://www.renalreg.org/publications-reports/.

[3] Canas L Lopez D Perez JF Bancu I Juega J Ariza A. Recurrent Glomerulonephritis in Renal Transplantation: Experience in Our Renal Transplantation Center. Transplant Proc (2015) 47(8):2354–6. doi: 10.1016/j.transproceed.2015.08.024 26518925

[4] Hariharan S Peddi VR Savin VJ Johnson CP First MR Roza AM. Recurrent and *De Novo* Renal Diseases After Renal Transplantation: A Report From the Renal Allograft Disease Registry. Am J Kidney Dis Off J Natl Kidney Foundation (1998) 31(6):928–31. doi: 10.1053/ajkd.1998.v31.pm9631835 9631835

[5] Allen PJ Chadban SJ Craig JC Lim WH Allen RDM Clayton PA. Recurrent Glomerulonephritis After Kidney Transplantation: Risk Factors and Allograft Outcomes. Kidney Int (2017) 92(2):461–9. doi: 10.1016/j.kint.2017.03.015 28601198

[6] Chailimpamontree W Dmitrienko S Li G Balshaw R Magil A Shapiro RJ. Probability, Predictors, and Prognosis of Posttransplantation Glomerulonephritis. J Am Soc Nephrol JASN (2009) 20(4):843–51. doi: 10.1681/ASN.2008050454 PMC2663828 19193778

[7] Briganti EM Russ GR McNeil JJ Atkins RC Chadban SJ. Risk of Renal Allograft Loss From Recurrent Glomerulonephritis. N Engl J Med (2002) 347(2):103–9. doi: 10.1056/NEJMoa013036 12110738

[8] Burton H Iyamu Perisanidou L Steenkamp R Evans R Mumford L Evans KM. Causes of Renal Allograft Failure in the UK: Trends in UK Renal Registry and National Health Service Blood and Transplant Data From 2000 to 2013. Nephrol Dialysis Transplant Off Publ Eur Dialysis Transplant Assoc - Eur Renal Assoc (2018) 34(2):355–64. doi: 10.1093/ndt/gfy168 29982787

[9] Pruthi R McClure M Casula A Roderick PJ Fogarty D Harber M. Long-Term Graft Outcomes and Patient Survival are Lower Posttransplant in Patients With a Primary Renal Diagnosis of Glomerulonephritis. Kidney Int (2016) 89(4):918–26. doi: 10.1016/j.kint.2015.11.022 26924061

[10] Saez-Rodriguez J Rinschen MM Floege J Kramann R. Big Science and Big Data in Nephrology. Kidney Int (2019) 95(6):1326–37. doi: 10.1016/j.kint.2018.11.048 30982672

[11] Nadkarni GN Coca SG Wyatt CM. Big Data in Nephrology: Promises and Pitfalls. Kidney Int (2016) 90(2):240–1. doi: 10.1016/j.kint.2016.06.003 27418085

[12] Sanchez-Pinto LN Luo Y Churpek MM. Big Data and Data Science in Critical Care. Chest (2018) 154(5):1239–48. doi: 10.1016/j.chest.2018.04.037 PMC6224705 29752973

[13] Ngiam KY Khor IW. Big Data and Machine Learning Algorithms for Health-Care Delivery. Lancet Oncol (2019) 20(5):e262–73. doi: 10.1016/S1470-2045(19)30149-4 31044724

[14] Cunningham H. GATE, a General Architecture for Text Engineering. Comput Humanities (2002) 36(2):223–54. doi: 10.1023/A:1014348124664

[15] Cunningham H Tablan V Roberts A Bontcheva K. Getting More Out of Biomedical Documents With GATE's Full Lifecycle Open Source Text Analytics. PloS Comput Biol (2013) 9(2):e1002854. doi: 10.1371/journal.pcbi.1002854 23408875 PMC3567135

[16] Broecker V Bardsley V Torpey N Perera R Montero R Dorling A. Clinical-Pathological Correlations in Post-Transplant Thrombotic Microangiopathy. Histopathology (2019) 75(1):88–103. doi: 10.1111/his.13855 30851188

[17] Frassinetti Fernandes P Ellis PA Roderick PJ Cairns HS Hicks JA Cameron JS. Causes of End-Stage Renal Failure in Black Patients Starting Renal Replacement Therapy. Am J Kidney Dis Off J Natl Kidney Foundation (2000) 36(2):301–9. doi: 10.1053/ajkd.2000.8974 10922308

[18] Ponticelli C Moroni G Glassock RJ. *De Novo* Glomerular Diseases After Renal Transplantation. Clin J Am Soc Nephrol CJASN (2014) 9(8):1479–87. doi: 10.2215/CJN.12571213 PMC4123406 24700797

[19] Cosio FG Cattran DC. Recent Advances in Our Understanding of Recurrent Primary Glomerulonephritis After Kidney Transplantation. Kidney Int (2017) 91(2):304–14. doi: 10.1016/j.kint.2016.08.030 27837947

[20] Sethi S Haas M Markowitz GS D'Agati VD Rennke HG Jennette JC. Mayo Clinic/Renal Pathology Society Consensus Report on Pathologic Classification, Diagnosis, and Reporting of GN. J Am Soc Nephrol JASN (2016) 27(5):1278–87. doi: 10.1681/ASN.2015060612 PMC4849835 26567243

[21] Moroni G Longhi S Quaglini S Rognoni C Simonini P Binda V. The Impact of Recurrence of Primary Glomerulonephritis on Renal Allograft Outcome. Clin Transplant (2014) 28(3):368–76. doi: 10.1111/ctr.12322 24757721

[22] Pippias M Stel VS Areste-Fosalba N Couchoud C Fernandez-Fresnedo G Finne P. Long-Term Kidney Transplant Outcomes in Primary Glomerulonephritis: Analysis From the ERA-EDTA Registry. Transplantation (2016) 100(9):1955–62. doi: 10.1097/TP.0000000000000962 26588008

[23] Lee BT Kumar V Williams TA Abdi R Bernhardy A Dyer C. The APOL1 Genotype of African American Kidney Transplant Recipients Does Not Impact 5-Year Allograft Survival. Am J Transplant (2012) 12(7):1924–8. doi: 10.1111/j.1600-6143.2012.04033.x PMC3387301 22487534

[24] Di Vico MC Messina M Fop F Barreca A Segoloni GP Biancone L. Recurrent IgA Nephropathy After Renal Transplantation and Steroid Withdrawal. Clin Transplant (2018) 32(4):e13207. doi: 10.1111/ctr.13207 29345747

[25] Barbour S Djurdjev O Gill JS Dong JJ Gill J. A Propensity Score Matched Analysis Shows No Adverse Effect of Early Steroid Withdrawal in non-Diabetic Kidney Transplant Recipients With and Without Glomerulonephritis. Kidney Int (2019) 96(2):460–9. doi: 10.1016/j.kint.2019.02.041 31248649

[26] Andresdottir MB Hoitsma AJ Assmann KJ Koene RA Wetzels JF. The Impact of Recurrent Glomerulonephritis on Graft Survival in Recipients of Human Histocompatibility Leucocyte Antigen-Identical Living Related Donor Grafts. Transplantation (1999) 68(5):623–7. doi: 10.1097/00007890-199909150-00005 10507479

[27] El-Zoghby ZM Stegall MD Lager DJ Kremers WK Amer H Gloor JM. Identifying Specific Causes of Kidney Allograft Loss. Am J Transplant (2009) 9(3):527–35. doi: 10.1111/j.1600-6143.2008.02519.x 19191769

[28] Stegall MD Park WD Dean PG Cosio FG. Improving Long-Term Renal Allograft Survival *via* a Road Less Traveled by. Am J Transplant (2011) 11(7):1382–7. doi: 10.1111/j.1600-6143.2011.03557.x 21564533

[29] Moroni G Longhi S Quaglini S Gallelli B Banfi G Montagnino G. The Long-Term Outcome of Renal Transplantation of IgA Nephropathy and the Impact of Recurrence on Graft Survival. Nephrol Dialysis Transplant Off Publ Eur Dialysis Transplant Assoc - Eur Renal Assoc (2013) 28(5):1305–14. doi: 10.1093/ndt/gfs472 23229925

[30] de Fijter JW. Recurrence of Glomerulonephritis: An Underestimated and Unmet Medical Need. Kidney Int (2017) 92(2):294–6. doi: 10.1016/j.kint.2017.04.007 28709599

[31] Uffing A Pérez-Sáez MJ Mazzali M Manfro RC Bauer AC de Sottomaior Drumond F. Recurrence of FSGS After Kidney Transplantation in Adults. Clin J Am Soc Nephrol CJASN (2020) 15(2):247–56. doi: 10.2215/CJN.08970719 PMC7015092 31974287

[32] Mulay AV van Walraven C Knoll GA. Impact of Immunosuppressive Medication on the Risk of Renal Allograft Failure Due to Recurrent Glomerulonephritis. Am J Transplant (2009) 9(4):804–11. doi: 10.1111/j.1600-6143.2009.02554.x 19353768

