# Supplementary material for: Incidence, Risk Factors, and Effect on Allograft Survival of Glomerulonephritis Post-transplantation in a United Kingdom Population: Cohort Study
Source: Front Nephrol. 2022 Jul 14;2:923813. doi: 10.3389/fneph.2022.923813 (PMC10479671; doi:10.3389/fneph.2022.923813)
Supplement: Supplementary file 1 [file DataSheet_1.docx]

Supplemental Appendix 1

Text-Mining Biopsy Software and its validation

Software to extract diagnoses of IgAN, FSGS, Membranous nephropathy, MPGN, TMA and Diabetic nephropathy from unstructured biopsy text reports was developed, using the open source General Architecture for Text Engineering (GATE)^16,17^ framework as a base.

Following a training process the software was validated comparing its output to results manually analysed by clinicians and showed a sensitivity of 100.0% (95% CI 88.1% - 100.0% for Centres 1, 2, 4 and 80.5% - 100.0% for Centre 3) and a specificity of 95.3% (95% CI 93.2% to 96.9%) for Centres 1, 2 and 4 and 88.5% (95% CI 83.0% to 92.8%) for Centre 3. Of note, all allograft biopsies presenting with GN attributed to secondary causes, based on histological characteristics, were not included in the study, leaving only possible cases of recurrence and *de novo* primary glomerulonephritis. More details on the training and validation process will be reported in a separate publication.
